# Supplementary material for: New Approaches for Calculating Moran’s Index of Spatial Autocorrelation
Source: PLoS One. 2013 Jul 12;8(7):e68336. doi: 10.1371/journal.pone.0068336 (PMC3709922; doi:10.1371/journal.pone.0068336)
Supplement: File S1 — A simple approach to calculating Moran’s index using MS Excel. (DOCX) [file pone.0068336.s001.docx]

## A simple approach to calculating Moran’s index using MS Excel

One of the four approaches to computing Moran’s index is illustrated in this Supporting Information file. Taking the capital cities of the 29 provinces, autonomous regions, and municipalities directly under the Central Government of China as an example (see the manuscript text), I will show how to calculate Moran’s index using the three-step method through Microsoft Excel. Before implementing a calculation, two datasets must be prepared as below: one is city population vector (Figure S1), and the other, spatial distance matrix (Figure S2).


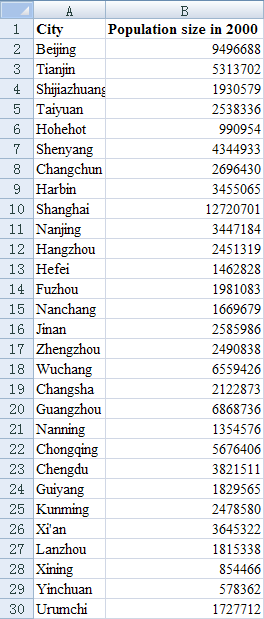


**Figure S1** The census data of the urban population of 29 Chinese cities in 2000


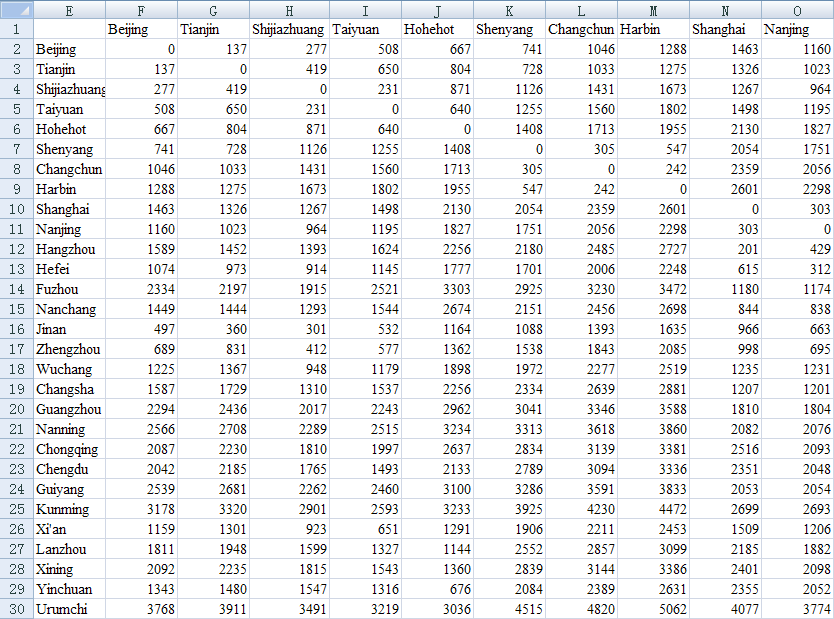


**Figure S2** Railroad distance matrix of 29 Chinese cities by railway (partial results)

### Step 1: Standardizing size measures

The procedure of data standardization is as below. First, compute the mean value of the city population using the MS Excel function “average”. In cell B31, input a formula such as “=AVERAGE(B2:B30)”, press **Enter**, and it will yield a mean about 3410661.31. Second, compute the population standard deviation (PSD) using the Excel function “stdevp”. In cell B32, input a formula such as “=STDEVP(B2:B30)”, press **Enter**, yield a PSD value around 2660468.49. Third, standardize the population size. Select a region in the worksheet including cells C2-C30, input a formula such as “=STANDARDIZE(B2:B30,B31,B32)”, press **Ctrl** and **Shift** and **Enter** at the same time, thus yield the standardized array indicative of the size vector (*z*), which is shown in Figure S3 (see File S2 for details).


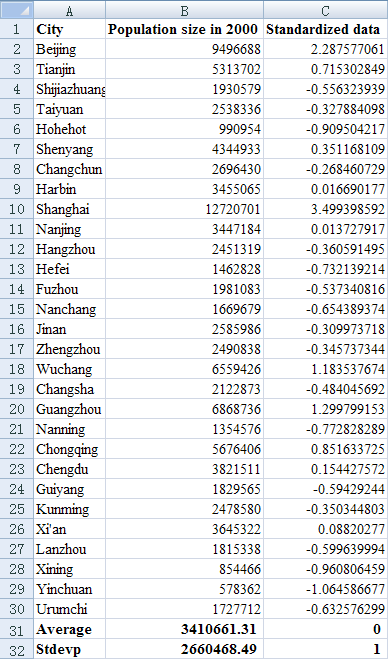


**Figure S3** The standardized data of the urban population of 29 Chinese cities

### Step 2: Generating spatial weights matrix

The process of yielding a spatial weights matrix is as follows. First, convert the spatial distance matrix into a spatial contiguity matrix. Select a spatial weight function such as

$v_{ij}=\left\{\begin{matrix} r_{ij}^{-1},\text{ }i\neq j \\ 0,\text{ }i=j \end{matrix}\right.$. (1)

In cell F33, input a formula such as “=IF(F2=0,"0",1/F2)”, press **Enter**, it will yield a number 0. Seize the bottom right corner of cell F33, drag it right and down, generate all the spatial contiguity values, which are shown in Figure S4. Second, summate the spatial contiguity values using the following formula:

$S=\sum_{i} \sum_{j} v_{ij}$, (2)

where *S* denotes an amount obtained as a of double summation. In cell AH62, input a formula “=SUM(F33:AH61)”, press **Enter**, yield a sum around 0.6296. Third, transform the spatial contiguity matrix into a spatial weights matrix. In cell F64, input a formula “=F33/$AH$62”, press **Enter**, and it will yield the first value of spatial weights. Catch hold of the bottom right corner of cell F64, pull it right and down, and produce all the values of the spatial weights matrix (*W*), which are displayed in Figure S5 (see File S2 for details).


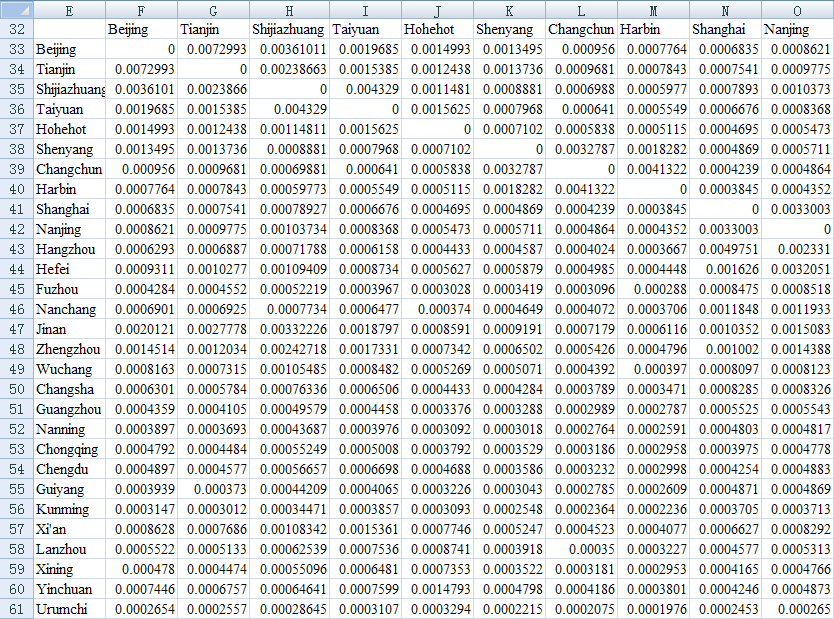


**Figure S4** Spatial contiguity matrix of 29 Chinese cities (partial results)


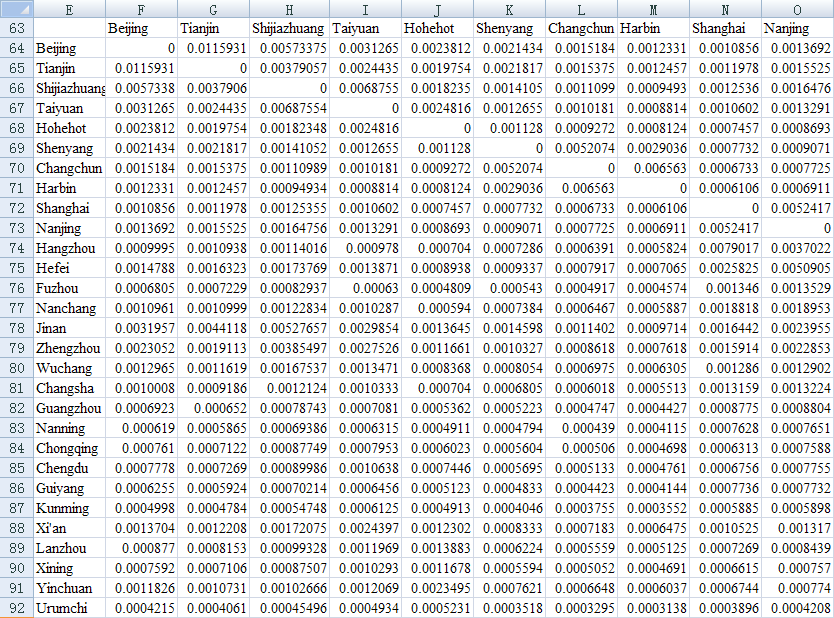


**Figure S5** Spatial weights matrix of 29 Chinese cities (partial results)

### Step 3: Computing Moran’s index

According to the formula *I*=*z*^T^*Wz*, it is easy to calculate Moran’s index (*I*) using Excel functions “mmult” and “transpose”. According to the number and result arrangement in the same worksheet, in any cell, say, B34, you can input a formula as below:

“=MMULT(MMULT(TRANSPOSE(C2:C30),F64:AH92),C2:C30)”

Pressing **Ctrl** and **Shift** and **Enter** at the same time yields the Moran’s index value immediately. The result is about -0.0315, which is based on PSD (see File S2 for details).

If we turn the PSD function, stdevp, into the sample standard deviation (SSD) function, stdev, we will have a revised value of Moran’s index. In other words, input a formula such as “=STDEV(B2:B30)” in cell B32 to replace the formula “=STDEVP(B2:B30)” yields a SSD value about 2707560.08. Accordingly, the PSD-based Moran’s index will change to the SSD-based Moran’s index, and the result is around -0.0304.
